# Vitamin Intake Reduce the Risk of Gastric Cancer: Meta-Analysis and Systematic Review of Randomized and Observational Studies

**DOI:** 10.1371/journal.pone.0116060

**Published:** 2014-12-30

**Authors:** Pengfei Kong, Qingqing Cai, Qirong Geng, Jing Wang, Yadong Lan, Youqing Zhan, Dazhi Xu

**Affiliations:** 1 State Key Laboratory of Oncology in South China, Collaborative Innovation Center for Cancer Medicine, Guangzhou, China; 2 Department of Gastric and Pancreatic Surgery, Sun Yat-sen University Cancer Center, Guangzhou, China; 3 Department of Medical Oncology, Sun Yat-sen University Cancer Center, Guangzhou, China; 4 Department of Hematology Oncology, Sun Yat-sen University Cancer Center, Guangzhou, China; 5 Department of Molecular Diagnosis, Sun Yat-sen University Cancer Center, Guangzhou, China; 6 Department of Oncological Surgery, the first affiliated hospital of Bengbu medical college, Bengbu, China; Rudjer Boskovic Institute, Croatia

## Abstract

**Aim:**

The association between vitamin intake and gastric cancer (GC) has been widely debated due to the relatively weak evidence. In this study, a meta-analysis of prospective and well designed observational studies were performed to explore this association.

**Methods:**

MEDLINE, Cochrane Library, and Sciencedirect were searched for studies of vitamin consumption and gastric cancer. This produced 47 relevant studies covering 1,221,392 human subjects. Random effects models were used to estimate summary relative risk (RR). Dose-response, subgroup, sensitivity, meta-regression, and publication bias analyses were conducted.

**Results:**

The RR of gastric cancer in the group with the highest vitamin intake was compared to that of the lowest intake group. Total vitamin intake was 0.78 (95% CI, 0.71−0.83). In 9 studies that individuals were given doses at least 4 times above the tolerable upper intake (UL) vitamins, the RR was 1.20 (95% CI, 0.99−1.44). However, in 17 studies that individuals received doses below the UL, the RR was 0.76 (95% CI, 0.68−0.86). Dose-response analysis was conducted on different increments in different types of vitamins (vitamin A: 1.5 mg/day, vitamin C: 100 mg/day, vitamin E: 10 mg/day) intake with a significant reduction in the risk of gastric cancer, respectively, 29% in vitamin A, 26% in vitamin C, and 24% in vitamin E.

**Conclusion:**

This meta-analysis clearly demonstrated that low doses of vitamins can significantly reduce the risk of GC, especially vitamin A, vitamin C, vitamin E.

## Introduction

Gastric cancer (GC) is the second leading cause of cancer-related mortality worldwide, with an estimated 989,600 new cases and accounted for 738,000 deaths in 2011. [1]. Despite the decrease in overall incidence, the total survival rate for GC patients did not improve significantly over the past two decades [2]. The only potentially curative treatment for GC is surgery, but only about 20–40% of patients can undergo radical resection. GC have become the main contributors to the total cancer burden in many parts of Asia [3]. Effective primary prevention strategies for GC, especially vitamin intake, have drawn considerable attention. For example, vitamins have been reported to play an important role in the prevention of GC in many studies [4], [5]. Some *in* *vitro* studies have also suggested that vitamins may prevent GC through different processes, such as scavenging the concentration of nitrite in the stomach, reducing oxidative stress, and inhibiting nitrosation.

Since 1970 s, the association between vitamin intake and GC has been assessed in a large and rapidly expanding body of literature.[6]–[8] However, most RCTs (Randomized, Placebo-Controlled Trials) included were not designed primarily to investigate the relationship between vitamins consumption and GC and performed in high-risk individuals. The current study is the first high-quality analysis of both prospective and retrospective studies to explore the relationship between vitamin intake and the riskof GC.

## Methods

### Search Strategy and Study Selection

MEDLINE, Cochrane Library and Sciencedirect were searched for studies of vitamin consumption and GC that were published only in English and performed on human participants from inception to February 2, 2014. Search terms were as follows: (vitamin OR supplement OR food OR diet OR dietary) AND (gastric OR stomach) AND (cancer OR neoplasm OR carcinoma). The reference lists of the articles identified were scanned manually for further potentially relevant studies. Authors were asked if they knew of any useful additional information (S1 Table and S2 Table in S1 File).

A study was included if it met the following criteria: 1) original article; 2) placebo-control, case-control or cohort design; 3) vitamin intake as the exposure of interest; 4) GC occurrence provided; 5) odds ratio (OR) or RR, and the corresponding 95% confidence interval (CI). Animal, mechanistic studies and non-peer-reviewed articles were excluded. This meta-analysis was performed in accordance with the Preferred Reporting Items for Meta-Analyses (PRISMA) statement checklist (checklist in checklist S1).

### Data Extraction and Quality Assessment

Four authors independently assessed the retrieved studies and extracted all data according to the pre-specified selection criteria. Disagreements were resolved by discussion. The following information was collected from each study: the last name of the first author, year of publication, study design, location, participant age, participant sex, study period, type of control subjects in case-control studies, sample size, type of vitamins evaluated and type of intake, the OR or RR with corresponding 95%CI for each category, and adjustments for confounders. When several articles discussed the same study, only the most recent or the one with the most complete data was included here. An evaluation system based on the Newcastle-Ottawa scale (NOS) was used to estimate the quality of observational studies. The studies included here were evaluated for three major factors: selection, comparability, and exposure/outcome assessment. The perfect score was 10 stars, and studies with 7 or more stars were defined as high-quality. Due to the risk of overestimation of beneficial intervention effects RCTs of low or inadequate methodological quality, we also assessed the RCTs methodological quality from the following domains: allocation sequence, allocation concealment, blinding, follow-up, and other apparent biases.

### Statistical Analysis

All analyses were performed with Rev Man version 5.2 and STATA 12.0. *P*<0.05 was defined as significant. ORs or RRs were extracted from the studies included here, and their standard errors (SEs) were calculated from their respective CIs. A random-effects model was used to quantify the relationship between vitamin intake and the risk of GC, considering both intra- and inter-study variability (τ^2^). The measure of effect of interest was RR with 95% CI. Because the absolute incidence of GC was low, the RR was mathematically similar to the OR in the studies included here. For this reason, all results were reported as RR for simplicity. Heterogeneity among studies was evaluated with*χ* ^2^and *I* ^2^ statistical testing. [9] To assess heterogeneity across all included studies, the variables of study design, geographic area, method of evaluation of vitamin intake, and dose were further examined in a meta-regression model. Subgroup stratification analyses were performed to assess variations in influence of these variables on overall results. Because the characteristics of the subjects, method of assessment of vitamin intake, and adjustments for confounders differed across studies, a sensitivity analysis was performed to assess any possible causes of heterogeneity and to evaluate the impact of different exclusion criteria on overall outcome. The influence of each single study on the results was evaluated by removing each study from consideration one at a time.

For the dose-response meta-analysis, only studies that listed the following data were analyzed: number of the case and control subjects, examined RR or OR and their 95% CI, and at least three quantitative exposure categories. For each included study, the mean vitamin intake for each quantitative exposure category was assigned an RR. Publication bias was assessed using funnel plots and Egger’s test method [10], [11].

## Results

### Search Results, Study Characteristics and Quality Assessment

**Figure 1:**
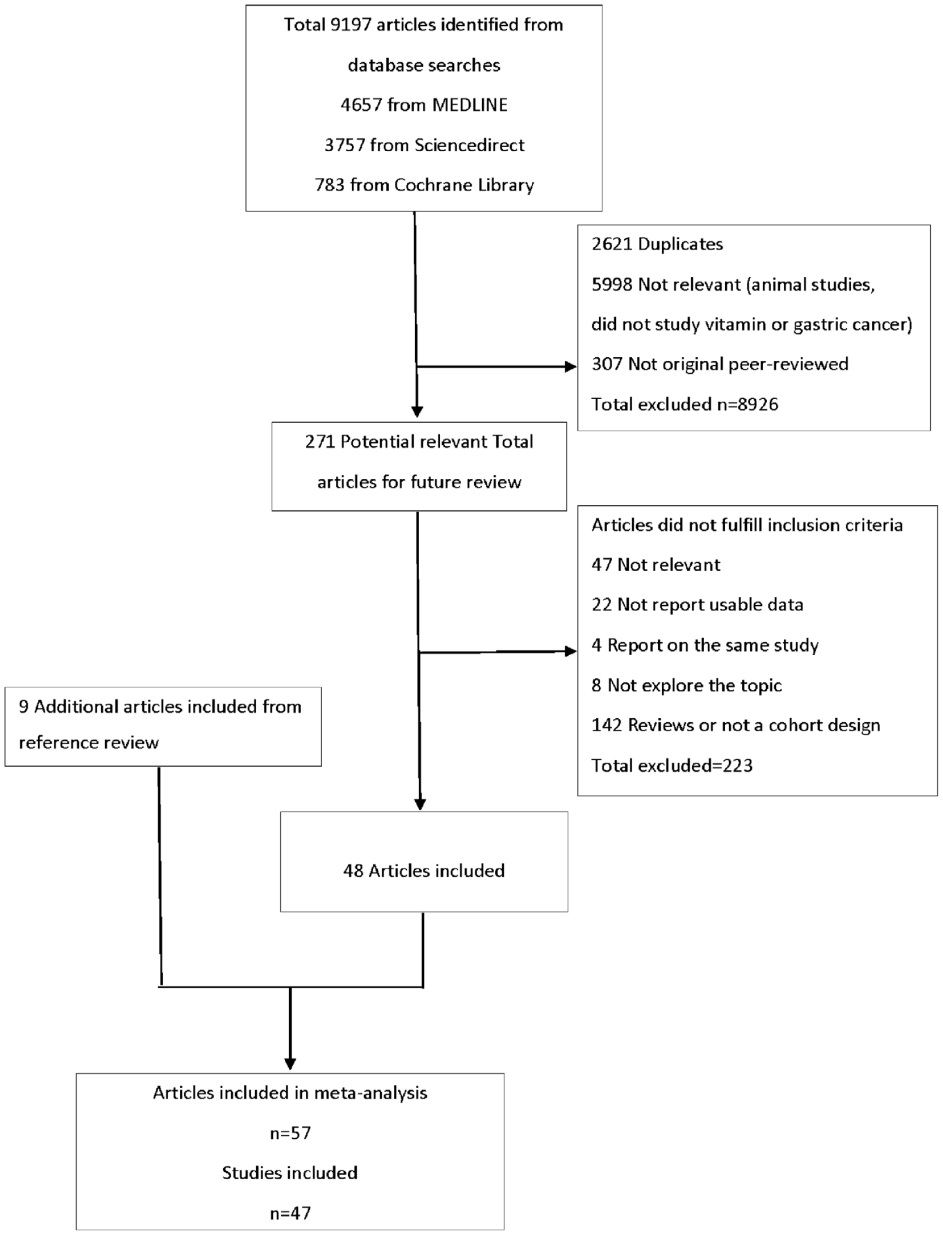
Flow Diagram of Study Selection.

A total of 47 studies published from 1985 to 2012 covering a total of 1,221,392 human participants, were identified in this meta-analysis (Fig. 1). Of the 47 studies (Table 1 and S3 Table in S1 File), 16 were population-based case-control (PCC) studies, [12]–[27] 13 were hospital-based case-control (HCC) studies, [6], [28]–[39] 11 were randomized placebo-controlled trials (RCTs), [7], [8], [40]–[57] and 7 were cohort studies.[5], [58]–[64] Sample sizes ranged from 216 [57] to 492,559. [59]. The number of GC cases varied from 2 [7] to 1124 [16]. Diagnosis of GC was based on histological findings in all studies.

**Table 1:** Characteristics of the included studies.

Author/year	Design	Country	Age (years)/sex	Study period	Total subjects	Number of events
Correa [6] 1985	HCC	United States	60–74 M/F	1979–1983	782	391
Risch [24] 1985	PCC	Canada	35–79 M/F	1979–1982	492	246
You [26] 1988	PCC	China	35–69 M/F	1984–1986	1695	564
Buiatti [11]1990	PCC	Italy	≤75 M/F	1985–1987	2175	1016
Boeing [27] 1991	HCC	Germany	32–80 M/F	1985–1988	722	143
NIT2 [40], [47], [53] 1993	RCT	China	40–69 M/F	1985–1991	3318	177
Ramon [23] 1993	PCC	Spain	30–80 M/F	1986–1989	351	117
Kabat [33] 1993	HCC	United States	60–70M/F	1981–1990	4666	122
Gonzalez [31] 1994	HCC	Spain	31–88M/F	1988–1989	708	354
Hansson [14] 1994	PCC	Sweden	40–79M/F	1989–1992	1017	338
La Vecchia [35] 1994	HCC	Italy	19–74M/F	1985–1992	2747	723
Cornee [28] 1995	HCC	France	66.6M/F	1985–1988	220	92
Zheng [63] 1995	Cohort	United States	55–69F	1986–1992	41837	26
PHS [38], [44] 1996	RCT	United States	40–84M	1982–1995	22071	41
Harrison [7] 1997	HCC	United States	54–62M/F	1992–1994	223	91
Ji [15] 1998	PCC	China	20–69M/F	1988–1989	2575	1124
Garcia-Closas [30] 1999	HCC	Spain	31–88M/F	1987–1989	708	354
Lopez-Carrilo [17] 1999	PCC	Mexico	20–98M/F	1989–1990	972	220
Terry [25] 2000	PCC	Sweden	66M/F	1995–1997	1073	258
De Stefani [29] 2000	HCC	Uruguay	30–89M/F	1997–1999	480	120
Correa [39], [50] 2000	RCT	Colombia	29–69M/F	–	976	2
Ekstrom [13] 2000	PCC	Sweden	67M/F	1989–1995	1732	567
Botterweck [57] 2000	Cohort	Netherlands	55–69M/F	1986–2003	120852	282
Mayne [19] 2001	PCC	United States	30–79M/F	1993–1995	1294	607
Palli [21] 2001	PCC	Italy	50–64M/F	1985–1987	943	382
Munoz [36] 2001	HCC	Venezuela	30–69M/F	1991–1997	777	292
Jedrychowski [32] 2001	HCC	Poland	–	–	340	80
HPS [43] 2002	RCT	United Kingdom	40–80M/F	1994–2001	20536	66
Chen [12] 2002	PCC	United States	70.3M/F	1986–1994	573	124
ATBC [37], [42], [49], [54], [55] 2003	RCT	Finland	50–69M	1985–1993	29133	249
Zhu [56] 2003	RCT	China	28–77M/F	1994–2001	216	5
Nomura [20] 2003	PCC	United States	26–95M/F	1993–1999	746	300
CARET [51] 2004	RCT	United States	45–69M/F	1985–1997	18314	35
SUVIMAX [45] 2004	RCT	France	35–60M/F	1994–2002	13017	4
Lissowska [16] 2004	PCC	Poland	50–70M/F	1994–1996	737	274
WHS [46] 2005	RCT	United States	54.6 F	1993–2005	39876	20
Qiu [22] 2005	PCC	China	28–85M/F	2000–2001	236	103
Kim [34] 2005	HCC	Korea	57.2M/F	1997–1998	272	136
SIT [41], [48] 2006	RCT	China	35–64M/F	1994–2003	3411	58
Lunet [18] 2006	PCC	Portugal	18–93M/F	2001–2004	544	233
Plummer [52] 2007	RCT	Venezuela	35–69M/F	1992–1999	1980	4
Larsson [60], [61] 2007	Cohort	Sweden	45–83M/F	1997–2005	82002	139
Carman [58] 2009	Cohort	United States	50–71M/F	1995–2003	492559	627
Pelucchi [8] 2009	HCC	Italy	22–80M/F	1997–2007	777	230
Neuhouser [62] 2009	Cohort	United States	50–79F	1993–2005	161808	101
Epplein [59] 2010	Cohort	China	40–74M/F	1996–2007	136442	338
Miyazaski [5] 2012	Cohort	Japan	>40M/F	1988–2002	2467	93

Abbreviations: F: female, M: male, FFQ: food frequency questionnaire, HCC: hospital-based case-control, PCC: population-based case-control, RCT: Randomized, Placebo-Controlled Trial.

Quality scores of observational study are summarized in S4 Table and S5 Table in S1 File. Quality scores ranged from 7 to 10. The average score was 8 for case-control studies and cohort studies. In this way, all observational studies were found to be high quality according to the NOS evaluation system. RCTs quality scores were also evaluated in S6 Table in S1 File. Twenty-two studies were excluded because they did not report usable data. Four papers were excluded because they reported the same study. Eight studies were excluded because they did not investigate the association between vitamin intake and GC risk. Non-cohort studies and 142 reviews were also excluded.

### Vitamin Intake and Risk of Gastric Cancer Risk

**Figure 2:**
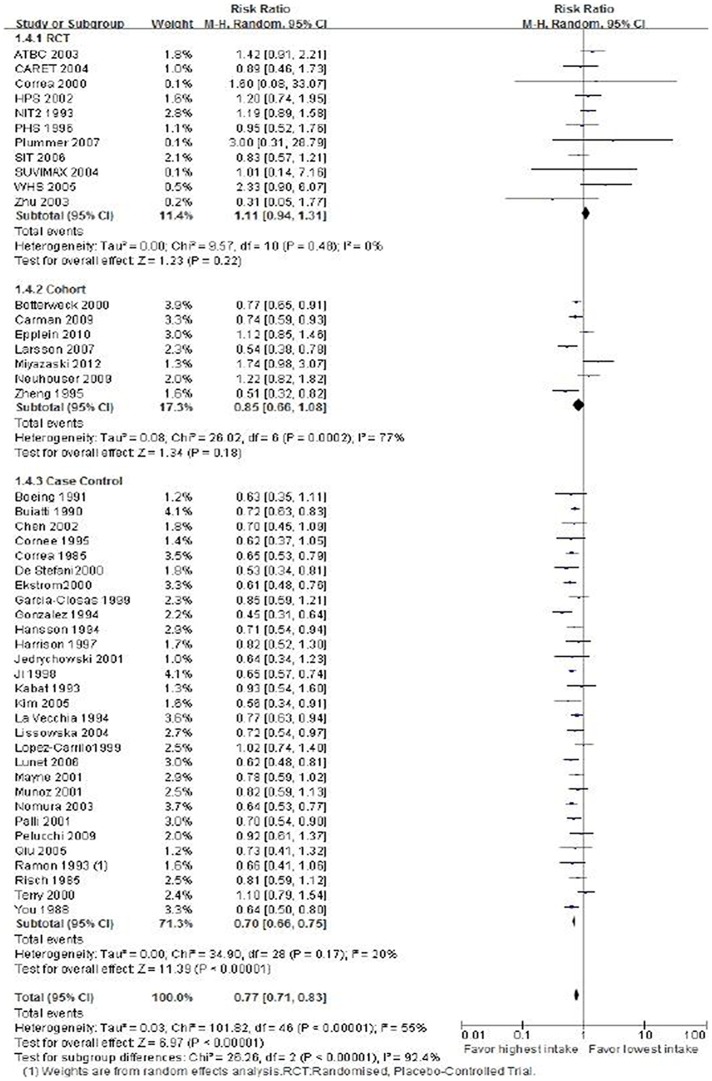
Forest plot of vitaminintake and risk of gastric cancer. Squares or diamonds to the left of the solid vertical line indicate benefit with vitamin intake.

A pooled analysis was performed on all 47 studies. The multivariable-adjusted RRs for each study and the combined RR for the highest versus the lowest categories of vitamin intake are presented in Fig. 2. Among all studies, 29 showed an inverse association between the vitamin intake and GC risk, [6], [12]–[17], [19]–[24], [27]–[30], [34], [36], [37], [41], [43], [46], [49], [52], [57]–[59], [61], [64] 15 of which were statistically significant. [6], [12], [14]–[16], [19], [21], [27], [30], [36], [37], [58], [59], [61], [64] In brief, a random effects model yielded a pooled RR for the highest vitamin intake group relative to the lowest vitamin intake group 0.77 (95% CI: 0.71–0.83). Significant heterogeneity was observed among studies (*P*<0.00001, *I* ^2^ = 55%). These results indicated that high vitamin consumption was associated with reduced GC risk.

### Dose-response Meta-analysis

**Figure 3:**
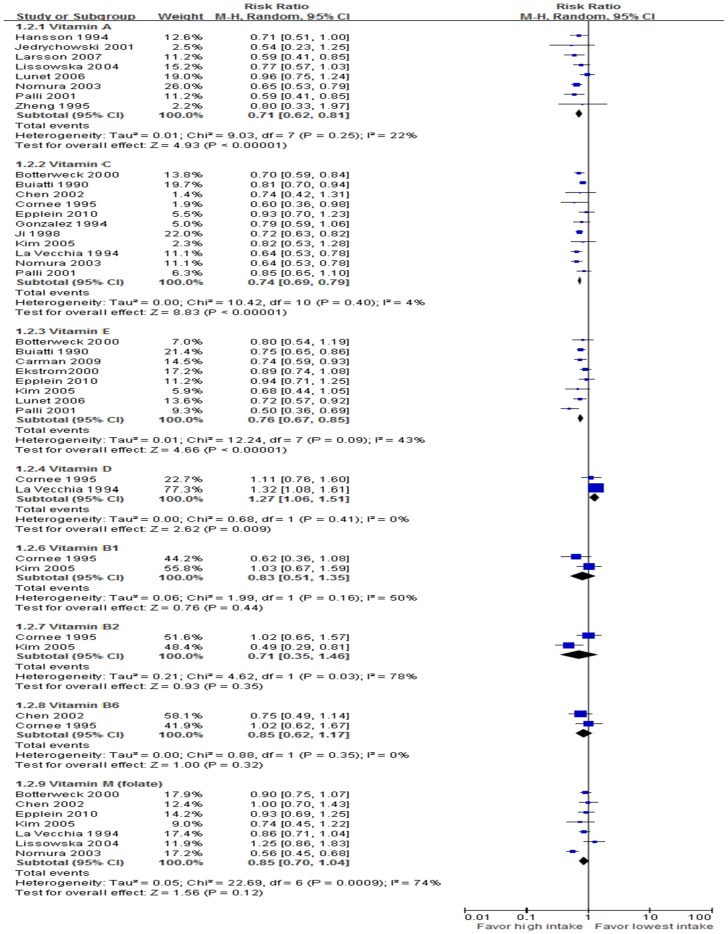
Forestplot of dose-response meta-analysis.

Eight studies that reported the RR and its 95% CI were included in the vitamin A dose-response meta-analysis. The summary RR for 1.5 mg/day (retinol equivalent) of vitamin A was 0.71 (95% CI, 0.62–0.81) without heterogeneity (*P*<0.00001, *I* ^2^ = 22%). Eleven studies that met the criteria were included in the vitamin C dose-response meta-analysis. The summary RR for 100 mg/day of vitamin C was 0.74 (95% CI, 0.69–0.79) without heterogeneity (*P*<0.00001, *I* ^2^ = 4%). Eight studies were qualified in the vitamin E dose-response meta-analysis. The summary RR for a 10 mg/day of dietary vitamin E intake was 0.76 (95% CI, 0.67–0.85) without heterogeneity (*P*<0.00001, *I* ^2^ = 43%). The rest results present in Fig. 3 and S7 Table in S1 File.

### Subgroup Analysis

#### 1). Study Design

Subgroup analysis by study design was performed. Significant inverse associations were observed in PCC studies (RR, 0.71; 95% CI, 0.66–0.76) and HCC studies (RR, 0.76; 95% CI, 0.68–0.85). Pooled analysis of RCTs showed no significant association with GC. Subgroup meta-analyses of 7 cohort studies showed a borderline significant decrease in GC to be associated with vitamin intake (RR, 0.85; 95% CI, 0.66–1.08) (Table 2).

**Table 2:** Subgroup analyses of vitamins intake and gastric cancer Risk.

			Heterogeneity test				
Group	NO. of reports	RR (95%)	χ^2^		P		I^2^(%)
Total	47	0.77(0.71,0.83)	101.82	<0.00001		55	
Design							
RCT	11	1.11(0.94,1.31)	9.57	0.22		0.0	
Cohort	7	0.85(0.66,1.08)	26.02	0.18		77	
Case-control	29	0.73(0.68,0.77)	37.64	<0.00001		26	
PCC	16	0.71(0.66,0.76)	19.51	<0.00001		23	
HCC	13	0.76(0.68,0.85)	15.71	<0.00001		24	
Geographic area							
East Asia	9	0.84(0.66,1.07)	36.39	<0.0001		78	
Europe	20	0.75(0.68,0.82)	35.38	<0.0001		46	
North America	14	0.79(0.69,0.90)	24.45	0.0004		47	
South America	4	0.71(0.48,1.06)	4.37	0.10		31	
Vitamin dose							
High dose	9	1.20(0.99,1.44)	6.72	0.06		0.0	
Low dose	17	0.76(0.68,0.86)	43.31	<0.00001		63	
Vitamin type							
Vitamin A	38	0.83(0.74,0.92)	130.46	0.0006		72	
Vitamin B	17	0.81(0.66,1.00)	111.42	0.06		86	
Vitamin C	37	0.66(0.59,0.73)	116.66	<0.00001		69	
Vitamin D	5	1.20(1.04,1.40)	2.07	0.01		0	
Vitamin E	32	0.75(0.67,0.85)	115.57	<0.00001		73	
Vitamin source							
plant	15	0.79(0.69,0.89)	36.96	0.002		65	
animal	11	0.78(0.68,0.89)	25.93	0.0003		61	
drug supplement	16	0.95(0.80,1.13)	33.09	0.58		55	
Lauren, s classification							
Diffuse	4	0.89(0.58,1.38)	3.98	0.60		25	
Intestinal	4	1.03(0.63,1.70)	11.93	0.89		75	
Location							
cardia	9	0.93(0.73,1.18)	18.97	0.55		58	
noncardia	9	0.94(0.71,1.24)	48.36	0.65		83	
Publication year							
<2000	18	0.77(0.69,0.84)	34.01	<0.00001		50	
≥2000	29	0.81(0.73,0.91)	64.09	0.002		58	
Sample size							
<1000	22	0.73(0.67,0.79)	25.09	<0.00001		16	
≥1000	25	0.84(0.75,0.94)	73.68	<0.00001		67	

Abbreviations: RCT: Randomized, Placebo-Controlled Trial, HCC: hospital-based case-control, PCC: population-based case-control.

#### 2). Geographic Area

Studies were stratified by geographic area, The RRs were 0.79 (95% CI, 0.69–0.90) for studies conducted in North America, [6], [13], [18], [20], [21], [25], [33], [35], [41], [46], [48], [52], [59], [63], [64] 0.75 (95% CI, 0.68–0.82) for studies in Europe. [12], [14], [15], [17], [19], [22], [24], [26], [28], [29], [31], [32], [34], [37], [39], [40], [44], [45], [47], [50], [55], [56], [58], [61] These results indicate a significant inverse association between vitamin intake and GC risk (Table 2).

#### 3). Vitamin Dose

**Figure 4:**
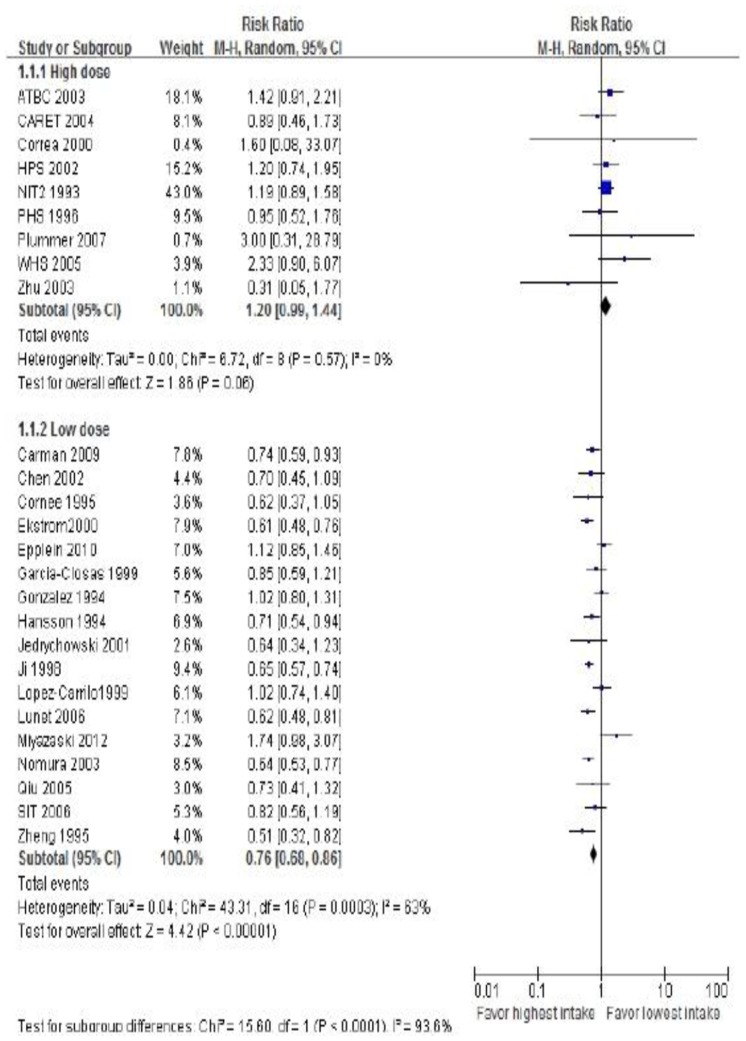
Forest plot of high dose vs low dose vitamin intake and risk of gastric cancer. Squares or diamonds to the left of the solid vertical line indicate benefit with vitamin intake.

Analysis by vitamin dose showed dosage (low dose) less than UL to be associated with lower risk of GC (Fig. 4). In 9 studies (n  = 152,848), individuals were given doses at least 4 times above the UL (high dose), and the RRs were 1.20 (95% CI, 0.99−1.44). Other individuals were given doses under the UL (low dose) in 17 studies (n  = 1,068,544). The RRs were 0.76(95% CI, 0.68−0.86) (Table 2). There was significant heterogeneity in low dose studies (*χ* ^2^ = 43.31; *P*<0.0001; *I* ^2^ = 63%), but not in high dose studies (*χ* ^2^ = 6.72; *P*  = 0.06; *I* ^2^ = 0.0%).

#### 4). Vitamin Type

Among subgroup analyses stratified by vitamin types, studies on vitamin A (RR, 0.83; 95% CI, 0.74–0.92) (vitamin A, retinol and beta-carotene were combined) [5], [7], [8], [12]–[18], [20]–[27], [29]–[31], [33]–[42], [44]–[47], [50]–[56], [58], [60], [61], [64] (RR, 0.83; 95% CI, 0.74–0.92), studies on vitamin B (all B group vitamins were combined) [8], [13], [16]–[18], [20], [21], [29], [32], [33], [36]–[39], [42], [54], [57], [58], [60] (RR, 0.81; 95% CI, 0.66–1.00), studies on vitamin C [6]–[8], [12]–[30], [32]–[39], [42], [43], [45], [47], [49], [51], [53], [54], [58], [60], [64] (RR, 0.66; 95% CI, 0.59–0.73), and studies in vitamin E [8], [12]–[26], [29], [33], [34], [36], [37], [39], [40], [42], [44], [45], [47], [48], [50], [53]–[56], [58]–[60], [64] (RR, 0.75; 95% CI, 0.67–0.85) produced similar results when the highest and lowest consumption categories were compared across vitamins (Table 2 and S1 Figure in S2 File).

#### 5). Vitamin Source

Among subgroup analyses stratified by the source of vitamin, relevant OR and RR and corresponding 95%CI for each category were extracted. The RRs were 0.79 (95% CI, 0.69–0.89) for plant vitamins,[6], [14], [16], [17], [19], [21], [24], [25], [27]–[29], [33], [35], [38], [60] 0.78(95% CI, 0.68–0.89) for animal vitamins, [6], [16], [17], [21], [25], [28], [29], [33], [35], [38], [64] and 0.95(95% CI, 0.80–1.13) for relevant drug supplement studies [7], [8], [26], [45]–[49], [52], [53], [56]–[59], [61], [63] (Table 2).

#### 6). Other

Subgroup stratification by location and Lauren’s classification, the no significant association was showed in cardia GC (RR, 0.93; 95% CI, 0.73–1.18) [5], [8], [14], [17], [20], [26], [35], [56], [59], non-cardia GC (RR, 0.94; 95% CI, 0.71–1.24) [5], [8], [13], [14], [17], , diffuse-type GC (RR, 0.89; 95% CI, 0.58–1.38) [5], [14], [33], [56]and in intestinal-type GC (RR, 1.03; 95% CI, 0.63–1.70). [5], [14], [33], [56](S2 Figure, S3 Figure, S4 Figure and S5 Figure in S2 File) However, significant associations were observed in the subgroup analysis by year of publication (before and after 2000) and sample size (<1000 and ≥1000) (Table 2).

### Sensitivity Analyses and Meta-regression

Sensitivity analyses were conducted to explore possible causes of heterogeneity and the effect of various exclusion criteria on the overall result were examined (data not shown). Sixteen studies that were not adjusted for total energy intake or dietary factors were omitted.[6]–[8], [13], [24], [26]–[28], [41]–[43], [45]–[47], [49], [51]–[54], [57], [58] The remaining studies produced an RR of 0.75 (95% CI, 0.69–0.82), with substantial evidence of heterogeneity (*P*<0.0001, *I* ^2^ = 59%). Restricting analysis to the 21 studies that were adjusted for smoking produced similar results (RR: 0.79, 95% CI: 0.71–0.89), but heterogeneity was still detectable (*P*<0.0001, *I* ^2^ = 52%).[6]–[8], [13], [14], [16]–[18], [20], [21], [23], [24], [26], [33], [35], [38], [39], [41], [42], [46]–[48], [51], [53], [54] Further exclusion of any single study did not change the overall results, which ranged from 0.77 (95% CI: 0.69–0.85) to 0.80 (95% CI: 0.72–0.88).

Meta-regression analysis demonstrated that study design (*P*  = 0.075), vitamin dosage (*P*  = 0.006), and method of assessing vitamin intake (*P*  = 0.006) were significant sources of heterogeneity. Study design alone explained 8.49% of the τ^2^ in the meta-regression analyses, vitamin dosage explained 24.54% of the τ^2^ and assessment of vitamins intake explained 23.84% (S8 Table in S1 File).

### Publication Bias

The funnel plot did not show any obvious asymmetry (S6 Figure in S2 File). No publication bias was detected using the Egger’s test (*P*  = 0.254).

## Discussion

In this study, data were available for more than 1.2 million individuals and more than 11,000 GC events. This work provided convincing evidence that vitamins intake is associated with a reduced risk of GC, especially at low doses. This relationship between vitamin intake and GC risk was apparent and consistent across a wide range of stratified subgroups. The dose-response meta-analysis indicated that appropriate increase vitamins intake (vitamin A: 1.5 mg/day, vitamin C: 100 mg/day, vitamin E: 10 mg/day) were associated with a statistically significant decreased risk of GC: 36% in vitamin A, 35% in vitamin C, and 32% in vitamin E, respectively.

In fact, since 1970 s, many observational studies and RCTs have evaluated the relationship between vitamin intake and the risk of GC, though results have been mixed. Zheng and Carman have provided evidence that higher vitamin intake may be relevant to the prevention of cancers of the upper digestive organs. [59], [64] A interesting study from China also reported higher circulating vitamin was associated with a reduced risk of incident GC [65]. However, Other investigators concluded that supplementation with vitamins has no major impact on the occurrence of GC [49], [55]. The discrepancy has several possible explanations, including differences in study design and type of vitamin intake (dietary or supplemental), differences in vitamin dosage used, differences in the assessment of vitamins intake and potential biases in each study. The lack of a statistically significant outcome in the clinical trials may have been caused by any of several methodological limitations of trials, such as short follow-up period and high levels of vitamins used.

Several meta-analyses of RCTs have also analyzed the effect of vitamins on the prevention of gastrointestinal cancer [66]–[69]. Wu revealed that vitamin A intake was inversely associated with GC risk by a meta-analysis, [66] while other researchers came to a opposite conclusion. They found that antioxidant vitamins supplements cannot prevent GC, and may even increase overall mortality [67]–[69]. However, there were many limitations in these meta-analyses. Firstly, the RCTs included in previous meta-analyses had higher doses than those usually found in individuals who ate a balanced diet, and some trials used dosages well above the recommended UL.[7], [40], [44], [45], [48], [50]–[52], [55], [56] (S9 Table in S1 File) The doses used in this study are more reasonable. Secondly, in prior articles, many retrospective case-control studies on this topic were excluded, despite which showed strongly that vitamins intake can prevent GC. In fact, most RCTs included in previous meta-analyses were not designed primarily to investigate the relationship between vitamins consumption and GC. This led to a lack of adjustment for the main confounders of GC. Moreover, most of these RCTs were performed in high-risk individuals, such as longtime smokers, [40], [44], [50], [55], [56] and subjects with a history of premalignant lesions, [8], [42], [54] which may not reflect the vitamin intake of normal risk population. Thus, the total number of subjects of previous meta-analyses was not very substantial and their conclusions should be treated with caution. This paper includes discussion of many well designed observational studies. These were conducted in normal risk populations, and are closely related to the topic. Indeed, it should not be assumed that RCTs always provide high-quality evidence for therapy. [70] High-quality observational studies are also important sources of powerful evidence in meta-analyses. [71].

Some studies have reported other non-antioxidant vitamins’ that affect GC prevention, [8], [33], [39], [54] others have focused on antioxidant vitamins (vitamin A, vitamin C and vitamin E). [45], [53], [56] However, in daily diet, it is difficult to draw distinctions between non-antioxidant vitamins and antioxidant ones. In this study, we combine them and demonstrate vitamins intake can reduce risk of gastric cancer.

The results of this meta-analysis indicate that relatively low doses of vitamins can prevent the occurrence of GC. Dose and method of administration are often clinically important and can be manipulated to prevent cancer [72]. For example, in the famous ATBC clinical trial, [56] the long-term use of vitamin A (4 years) at a high dose (7.5 mg/day, about 2.5 times the UL) showed no benefit with respect to preventing lung cancer in high-risk individuals (smokers). However, in a HCC study conducted in southwestern France, the author emphasized that dietary vitamin A (2 mg/day, less than the UL) might have a distinct and important protective effect on lung cancer prevention. [73] Some high-quality retrospective analyses indirectly showed that relatively low doses of vitamins (less than UL) prevented cancer more effectively. [74] These conclusions are similar to our study. Notably, in the dose-response analysis, we revealed that relatively low doses vitamin A, vitamin C, and vitamin E can significantly reduce the risk of GC (vitamin A: 1.5 mg/day, vitamin C: 100 mg/day, vitamin E: 10 mg/day). They are hopeful to be a possible recommendation dosage of vitamin intake for GC prevention. However, the mechanism of low doses of vitamins reduce risk of cancer is still unknown. Some researchers have also revealed that the long term administration of mega-dosages of vitamins can bring out many adverse effects.

The current study also draws attention to the fact that vitamins from food (plant or animal) contribute more to reductions in GC risk than synthetic vitamin supplements. Some investigators have noted that the bioavailability of vitamins differs depending on whether the vitamin comes from food or is synthetic, which could explain the results. For example, Carr reported differences in bioavailability between synthetic and kiwifruit-derived vitamin C in a randomized crossover pharmacokinetic study [75].

Subgroup analyses by vitamin types, vitamin A, vitamin B, vitamin C and vitamin E produced similar outcomes, but vitamin D did not. Vitamin D is not really a vitamin. It is the precursor to the steroid hormone calcitriol and play an important role in determining cancer risk [76]. Accumulating results from preclinical and clinical studies strongly suggest that vitamin D deficiency increases the risk of developing cancer. Vitamin D supplements might be an economical and safe way to reduce the incidence of cancer and improve cancer prognosis and outcome. However, in the current meta-analysis, only 5 case-control studies have explored the association between vitamin D and GC risk [8], [20], [29], [37], [39]. This might be the reason for the discrepancy.

During the past 3 decades, many studies have reported that the mechanisms of different types of vitamins may reduce the risk of GC. This includes vitamin that function in an irreversibly oxidized form, vitamins that reduce the concentration of nitrite in the stomach, and vitamins that affect free radical-mediated damage to the stomach epithelium [75]. In addition, some studies have indicated that vitamin E is a potent lipid-soluble antioxidant and might be involved in GC prevention by reducing oxidative stress [77].

### Study Strengths and Limitations

The current study has several strengths. First, it addresses both non-antioxidant and antioxidant vitamins and covers a large number of human subjects (1,221,392). This increased the statistical power of the analysis considerably. Second, these results are less likely to be explained by recall and selection bias because of the inclusion of 18 prospective studies (11 RCTs and 7 cohort studies). Third, a statistically significant association was observed in most of the subgroups that adjusted for confounders. These subgroups produced results similar to those of other subgroups. Fourth, the current study not only included RCTs but also many other high-quality observation studies. This was beneficial to identify the relationship between vitamins and GC. Fifth, a significant dose-response relationship was observed between vitamin intake and GC risk (Table 2). Finally, this is the first study to discuss the influence of dosage in the relationship and the effect of all kinds of vitamin compare with early studies.

Several limitations should be addressed in this study. First, the studies included in this article have been conducted in different countries since the 1980 s, but some studies have had faulty designs, were not designed primarily to study vitamins consumption, and lacked stratification. This makes the combination of these studies with a random-effects model problematic. The second limitation is that the quality and power of any meta-analysis are dependent on the quality and comparability of data from the included studies. The analysis would be more convincing if original data were available, making an adjustment estimate possible. We have attempted to contact the authors of original studies to obtain more detailed information. However, it is very difficult to obtain all the original data regarding published studies. Third, the range of vitamin taken in by individuals with the lowest vitamin intake and those with the highest differed among the studies, which caused heterogeneity in the pooled analysis. Fourth, there were relatively few eligible studies of the dose-response analysis. These studies contained a few cohort and case-control studies. More and more in-depth studies are necessary.

### Implications

The current findings may have several implications. First, vitamin intake can reduce the risk of GC, but excessive and long-term intake might disturb this anti-tumor function. Second, dietary vitamins might prevent GC more effectively than supplements. Third, according to the results of the current meta-analysis, overall vitamin intake can reduce the risk of GC by 23%. This reduction could be translated into a decrease of as many as 169,740 GC deaths and 227,608 new cases per year worldwide [1]. Last, the desired low but sufficient level of vitamin intake may be achieved by fruit and vegetable consumption. This is consistent with results indicating fruit and vegetable intake is inversely associated with the incidence of GC [78].

## Conclusions

In summary, unlike early studies, this article conducted well designed observational studies which conducted in normal risk populations and discuss the influence of dosage in the relationship and the effect of all kinds of vitamins. It shows clearly that low doses of vitamins can significantly reduce the risk of GC, especially vitamin A, vitamin C, vitamin E. However, because of potential bias and confounding factors, these results should be treated with caution. More and better-designed large clinical trials should use appropriate doses of vitamins in order to generate a more visible association between vitamin intake and the risk of GC.

## Supporting Information

S1 PRISMA Checklist **Preferred Reporting Items for Meta-Analyses (PRISMA) statement checklist.**
(DOC)

S1 File **Supporting Information Tables.** S1 Table Search strategy in PubMed and Cochrane Library. S2 Table. Search strategy in Sciencedirect. S3 Table. Characteristics of the included studies. S4 Table. Methodological quality of case-control studies included in the meta-analysis. S5 Table. Methodological quality of cohort studies included in the meta-analysis. S6 Table. Methodological quality of RCTs included in the meta-analysis. S7 Table. Dose-response analysis. S8 Table. Meta-regression analysis. S9 Table. Tolerable upper intake levels of vitamins.
(DOCX)

S2 File **Supporting Information Figures.** S1 Figure. Subgroup analysis: Forest plot of vitamin type. CI, confidence interval; df, degrees of freedom; I2, the percentage of total variation across studies that is caused by heterogeneity rather than by chance Squares or diamonds to the left of the solid vertical line indicate benefit with each type of vitamin intake; this is conventionally significant (P<0.05) only if the horizontal line or diamond does not overlap the solid vertical line. Relative risks are analysed with random-effects model. S2 Figure. Subgroup analysis: Forest plot of Lauren’s classification (intestinal). CI, confidence interval; df, degrees of freedom; I^2^, the percentage of total variation across studies that is caused by heterogeneity rather than by chance. S3 Figure. Subgroup analysis: Forest plot of Lauren’s classification (diffuse). CI, confidence interval; df, degrees of freedom; I^2^, the percentage of total variation across studies that is caused by heterogeneity rather than by chance. S4 Figure. Subgroup analysis: Forest plot of location (cardia). CI, confidence interval; df, degrees of freedom; I^2^, the percentage of total variation across studies that is caused by heterogeneity rather than by chance. Relative risks are analysed with random-effects model. S5 Figure. Subgroup analysis: Forest plot of location (non-cardia). CI, confidence interval; df, degrees of freedom; I^2^, the percentage of total variation across studies that is caused by heterogeneity rather than by chance. Relative risks are analysed with random-effects model. S6 Figure. Funnel plot of included studies. RCT: Randomized, Placebo-Controlled Trial. The oblique line in the center is the natural logarithm of pooled relative risk, and the 2 solid lines are pseudo 95% confidence limits.
(DOCX)
