# Supplementary material for: Vitamin Intake Reduce the Risk of Gastric Cancer: Meta-Analysis and Systematic Review of Randomized and Observational Studies
Source: PLoS One. 2014 Dec 30;9(12):e116060. doi: 10.1371/journal.pone.0116060 (PMC4280145; doi:10.1371/journal.pone.0116060)
Supplement: S1 File — Supporting Information Tables. S1 Table Search strategy in PubMed and Cochrane Library. S2 Table. Search strategy in Sciencedirect. S3 Table. Characteristics of the included studies. S4 Table. Methodological quality of case-control studies included in the meta-analysis. S5 Table. Methodological quality of cohort studies included in the meta-analysis. S6 Table. Methodological quality of RCTs included in the meta-analysis. S7 Table. Dose-response analysis. S8 Table. Meta-regression analysis. S9 Table. Tolerable upper intake levels of vitamins. (DOCX) [file pone.0116060.s002.docx]

**File S1**

| **S1 Table.** Search strategy in PubMed and Cochrane Library. |
| --- |
| **S2 Table.** Search strategy in Sciencedirect. |
| **S3 Table.** Characteristics of the included studies |
| **S4 Table.** Methodological quality of case-control studies included in the meta-analysis |
| **S5 Table.** Methodological quality of cohort studies included in the meta-analysis |
| **S6 Table.** Methodological quality of RCTs included in the meta-analysis. |
| **S7 Table.** Dose-response analysis. |
| **S8 Table.** Meta-regression analysis. |
| **S9 Table.** Tolerable upper intake levels of vitamins. |

# **S1 Table.** Search strategy in PubMed and Cochrane Library.

| 1. Vitamin |
| --- |
| 2. Intake |
| 3. Supplement |
| 4. Food |
| 5. Diet |
| 6. Dietary |
| 7. 1 OR 2 OR 3 OR 4 OR 5 OR 6 |
| 8. Gastric |
| 9. Stomach |
| 10. 8 OR 9 |
| 11. Cancer |
| 12. Neoplasm |
| 13. Carcinoma |
| 14. Adenocarcinoma |
| 15. Turmor |
| 16. 11 OR 12 OR 13 OR 14 OR 15 |
| 17. 7 AND 10 AND 16 |

**S2 Table.** Search strategy in Sciencedirect.

| 1. Gastric. Ti, ab. |
| --- |
| 2. Stomach. Ti, ab. |
| 3. 1 OR 2 |
| 4. Vitamin. Ti, ab. |
| 5. Diet. Ti, ab. |
| 6. Dietary. Ti, ab. |
| 7. 4 OR 5 OR 6 OR 7 |
| 8. Intake. Ti, ab. |
| 9. Supplement. Ti, ab. |
| 10. 9 OR 10 |
| 11. Cancer. Ti, ab. |
| 12. Turmor. Ti, ab. |
| 13. Neoplasm. Ti, ab. |
| 14. Carcinoma. Ti, ab. |
| 15. Adenocarcinoma. Ti, ab. |
| 16. 12 OR 13 OR 14 OR 15 OR 16 |
| 17. 3 AND 8 AND 11 AND 17 |

**S3 Table.** Characteristics of the included studies

| **Author**  **/year** | **Assessment of**  **intake/food item number/vitamins intake calculation/nutrient database** | **Time period of dietary questions or average follow-up period**  **(years)** | **Intervention vitamins items**  **and average intake comparison** | **Adjusted or matched variables** |
| --- | --- | --- | --- | --- |
| Correa6` 1985 | Dietary history questionnaire/59/amou nt× frequency/USDA's food composition tables | — | Vitamin A,C Quartile:(Ⅳ VSⅠ) | Sex,  socio-econo mic status, and smoking |
| Risch24 1985 | FFQ/—/amount× frequency/USDA's food | 1 year before | VitaminA:9.1mg/d VS 0.9mg/d, | Age, sex, and area |

composition tables interview Vitamin B1:6.0mg/d VS

0.7mg/d,

Vitamin B2:7.9mg/d VS 1.1mg/d,

Vitamin C:465mg/d VS 39mg/d,

Vitamin E:170mg/d VS 13.7mg/d

You26 1988

Buiatti11 1990

Boeing27 1991

FFQ/85/portion size×

frequency/Chinese food composition tables

FFQ/146/portion size× frequency/Italian food tables

FFQ/74 /portion size× frequency/German food tables

20 years before interview

12-month

—2 years before interview

1. years before interview

Vitamin A(Retinol), C Quartile:(4 VS 1)

Vitamin A(Beta-carotene, Retinol):6.6mg/d VS 1.6mg/d,

Vitamin C:149mg/d VS 35mg/d, Vitamin E:11mg/d VS 5mg/d

Vitamin C Quartile:(Ⅴ VSⅠ)

Age, sex, and income

Age, sex,

area, place of residence, socio-econo mic status, family history, and energy intake

Age, sex, and area

NIT240,47,53 1993

—/—/ The amount of daily oral/—

follow-up about 6 years

Vitamin A (3.4mg/d),B1 (5 mg/d),B2 (5.2 mg/d),B6

(6mg/d),B12 (18 μg/d),C

(180 mg/d),D (20μg/d),E (60 mg/d), folate (800 μ

g/d) VS Placebo

Age, sex, smoking, and alcohol use

Ramon23 1993

FFQ/89/ amount×

frequency/ Spanish food composition tables

1. months before interview

Vitamin A,C,E Quartile:(4 VS 1)

Age, sex, education, smoking, and alcohol use

Kabat33 1993

FFQ/30/fiber value/ USDA's food

composition tables

5 years before interview

Vitamin A ,C Quartile:(4 VS 1)

Age, smoking, alcohol use, education, area, and dietary factor

| Gonzalez31 1994 | FFQ/77/ amount× frequency/ Spanish and British food  composition tables | 1 year before interview | Vitamin C:140mg/d VS 64mg/d | energy intake |
| --- | --- | --- | --- | --- |
| Hansson14 1994 | FFQ/45/ portion size× frequency/Swedish food composition tables | Adolescen ce and 20 years before interview | Vitamin A( Beta-carotene, Retinol),C, E  Quartile:(4 VS 1) | Age, sex, and energy intake |
| La Vecchia35 1994 | FFQ/29/portion size× frequency/Italian food tables | 1 year before interview | Vitamin A(Beta-carotene, Retinol):10.5mg/d VS 3.2 mg/d, Vitamin C:157mg/d VS 79 mg/d, Vitamin E:6.26mg/d VS 3.87mg/d,  Folate:261μg/d VS 162μ  g/d | Age, sex, education, family history, BMI, and energy intake |
| Cornee28 1995 | Dietary history questionnaire/—/ portion size  ×frequency/French and English food tables | 1 year before interview | Vitamin A:1.3mg/d VS 0.9mg/d, Vitamin B1:1.3mg/d VS 0.9mg/d, Vitamin B2:1.7mg/d VS 1.2mg/d,  Vitamin B6:1.2mg/d VS 0.9mg/d, Vitamin C: 116mg/d VS 70mg/d, Vitamin D:1.4mg/d VS 0.9mg/d, Vitamin E:3.8mg/d VS 2.7mg/d | Age, sex, occupation and energy intake |
| Zheng63 1995 | FFQ/127/frequency  ×portion size/— | follow-up about 6 years | Vitamin A(Retinol): ＞  1.7mg/d VS ＜0.6mg/d,  Vitamin C: ＞5.56mg/d VS  ＜4.97mg/d ,  Vitamin E: ＞ 2.93 mg/d VS ＜2.01mg/d | Age, smoking, and energy intake |
| PHS38,44 1996 | FFQ/Beta-carotene, aspirin, placebo/ The amount of daily oral/— | follow-up about 7 years | Vitamin A (Beta-carotene 25 mg/d) VS Placebo | Age, smoking, alcohol use, and BMI |

| Harrison7 1997 | FFQ/60/frequency  ×portion size/USDA's food composition tables | 1 year before interview | Vitamin A,B1,B2,B6,C,E,folate  Quartile:(Ⅳ VSⅠ) | Age, sex, race, energy intake, education, smoking, alcohol use, and BMI |
| --- | --- | --- | --- | --- |
| Ji15 1998 | FFQ/84/ amount× Frequency/Chinese food composition tables | 10 years before interview | Vitamin A(Carotene),B2,C,E Quartile:(4 VS 1) | Age, income, education, smoking, alcohol use and energy intake |
| Garcia-Clos as30  1999 | Dietary history questionnaire  /77/portion size  ×frequency/Spanish food composition tables | 1 year before interview | Vitamin  A(Beta-carotene):2.8mg/d VS 0.5mg/d | Energy intake, and dietary factor |
| Lopez-Carril o17  1999 | FFQ/70/portion size  ×frequency/USDA's food composition tables | 1 year before interview | Vitamin A: ≥1.1mg/d VS  0.5mg/d≤,  Vitamin C: ≥392mg/d VS 186mg/d≤,  Vitamin E: ≥13mg/d VS 8.4mg/d≤,  Folate: ≥ 466 μ g/d VS  257μg/d≤ | Age, sex, energy intake,  socio-econo mic status, smoking, salt use, medical history |
| Terry25 2000 | FFQ/63/frequency× nutrient content/Swedish food composition tables | 20 years before interview | Vitamin A:5.0mg/d VS 0.7mg/d,  Vitamin C:88mg/d VS 29mg/d,  Vitamin E:6.8μg/d VS 5.5  μg/d | Age, sex,  BMI, and smoking |
| De Stefani29 2000 | FFQ/64/portion size  ×frequency/Spanish food composition tables | 2 years before interview | Vitamin A (Beta-carotene):  ＞5mg/d VS ≤2.5mg/d | Age, sex,  area, and energy intake, |
| Correa39,50 2000 | —/Beta-carotene, vitamin C, placebo/ The amount of daily oral/— | follow-up about 6 years | Vitamin A（Beta-carotene 30mg/d）, Vitamin C (1g/d)  VS Placebo | Age, sex, smoking, alcohol use, and BMI |

| Ekstrom13 2000 | FFQ/45/frequency× nutrient content/Swedish food composition tables | 20 years before interview | Vitamin A(Beta-carotene):  ≥2.7mg/d VS 1.1mg/d≤, Vitamin C: ≥65.5mg/d VS 33.2mg/d≤,  Vitamin E: ≥11.1mg/d VS 4.0mg/d≤ | Age, sex, socio-econo mic status, energy intake, smoking, dietary factor, and urban environment |
| --- | --- | --- | --- | --- |
| Botterweck  57  2000 | FFQ/150/portion size× frequency/Dutch food composition tables | follow-up about 6.3 years | Vitamin A(Retinol, Beta-carotene): 5.6mg/d VS 1.7mg/d, Vitamin C:134.8mg/d VS 55mg/d, Vitamin E:21.7mg/d VS 6.59mg/d, folate:384mg/d VS 202mg/d | Age , sex; smoking, alcohol use, and BMI |
| Mayne19 2001 | FFQ/104/portion size× frequency/US nutrition coding center nutrient data system | 3-5 years before interview | Vitamin A(Beta-carotene, Retinol),B1,B2,B6,B12,C,D,  E,folate High VS Low | Age, sex,  area, race, income, education, BMI,  smoking, alcohol use, and energy intake |
| Palli21 2001 | FFQ/181/ portion size× frequency/Italian food composition tables | 1 year before interview | Vitamin A(Beta-carotene, Retinol):5.1mg/d VS 2.0mg/d, Vitamin C:142mg/d VS 56mg/d,  Vitamin E:10mg/d VS  5.5mg/d | Age, sex, socio-econo mic status, family history, area, BMI , energy intake and dietary factor |
| Munoz36 2001 | FFQ/75/ portion size× frequency/Colombian food composition tables | 15 to 20 years before interview | Vitamin A  (Retinol),B1,B2,B6,B12,C,fo  late  Quartile:(4 VS 1) | Age, sex, alcohol use, smoking, socio-econo mic status,  and energy |

| intake | | | | |
| --- | --- | --- | --- | --- |
| Jedrychows ki32 2001 | FFQ/148/ portion size× frequency/Polish food composition tables | 1 to 5 years before interview | Vitamin A(Retinol): ≥ 1.24mg/d VS ＜0.64mg/d, Vitamin C: ≥ 66.05mg/d VS ＜66.05mg/d,  Vitamin E: ≥ 10.05mg/d VS ＜7.75mg/d | Education, energy intake, dietary factor and physical activity |
| HPS43 2002 | —/Beta-carotene, vitamin C, E, placebo/ The amount of daily oral/— | follow-up about 7 years | Vitamin A（Beta-carotene 20mg/d）, Vitamin C (250  mg/d), Vitamin E (600 mg/d) VS Placebo | Age, sex, alcohol use, and smoking |
| Chen12 2002 | Health habits and history questionnaire/60/porti on size × frequency/ USDA's food  composition tables | 1 year before interview | Vitamin A(Beta-carotene, Retinol),B2,B6, C,E, folate Quartile:(4 VS 1) | Age, sex, respondent type, alcohol use, smoking, education, and family history |
| ATBC37,42,49, 54,55  2003 | Dietary history questionnaire/Beta-car otene, vitamin E, placebo/ The amount of daily oral/— | follow-up about 6.1 years | Vitamin A（Beta-carotene 20mg/d）, E (50mg/d) VS  Placebo | Age, smoking, alcohol use, energy intake, and BMI |
| Zhu56 2003 | —/—/ The amount of daily oral/— | follow-up about 7 years | Vitamin A（Beta-carotene  30mg/d）, folate (20mg/d) VS Placebo | Age, sex |
| Nomura20 2003 | FFQ/>250/portion size  ×frequency/USDA's food composition tables, food data bank of the National Cancer Institute of Canada | 1year before interview | Vitamin A(Beta-carotene), C,E, folate  Quartile:(3 VS 1) | Age, race, smoking, education, medical history, NSAID use, family history and energy intake |

CARET51 2004

—/—/ The amount of daily oral/—

follow-up about 12 years

Vitamin A(Beta-carotene 30mg/d, Retinol 7.5 mg/d) VS Placebo

Age, sex,

race, and smoking

SUVIMAX45 2004

—/—/ The amount of daily oral/—

follow-up about 7 years

Vitamin A（Beta-carotene 6mg/d ） , C (120mg/d),E

(30mg/d) VS Placebo

Age, education, smoking, postmenopa usal status and BMI

Lissowska16 2004

WHS46 2005

FFQ/118/portion size× frequency/US and Polish food tables

Health questionnaire/—/ The amount of daily oral/—

4 years before interview

follow-up about 9 years

Vitamin A(Beta-carotene, Retinol):3.5mg/d VS 1.2mg/d,

Vitamin C:10mg/d VS 4.3mg/d,

Vitamin E:10.6mg/d VS 4.5mg/d, folate:400 μ g/d VS 220μg/d

Vitamin E (200mg/d) VS Placebo

Age, sex, education, smoking, and energy intake

Age, smoking, alcohol use, and physical activity, postmenopa usal status,

BMI and energy intake

Qiu22 2005

Kim34 2005

FFQ/109/portion size× frequency/Chinese food composition tables

FFQ/>60/portion size× frequency/Korean Foods and Nutrients

1 year before interview

12-month

—3 years before

Vitamin A(Carotene),C,E Quartile:(4 VS 1)

Vitamin A(Beta-carotene, Retinol):6.0mg/d VS 3.0mg/d, Vitamin

Age, area education, socio-econo mic status, smoking, alcohol use and energy intake

Age, sex, socio-econo mic status,

Database interview B1:1.7mg/d VS 1.3mg/d,

Vitamin B2:1.7mg/d VS 1.2mg/d,

Vitamin B6:2.8mg/d VS 1.9mg/d, Vitamin C:160mg/d VS 90mg/d, Vitamin E:12.5mg/d VS

8.0mg/d, folate: 340μg/d

VS 210μg/d

family history, and Helicobacter pylori infection

SIT41,48 2006

Lunet18 2006

—/—/ The amount of daily oral/—

FFQ/82/portion size× frequency/Portuguese food composition tables

follow-up about

14.7 years

1 year before interview

Vitamin C (250 mg/d), Vitamin E (100mg/d) VS Placebo

Vitamin C: ≥ 144.7mg/d VS≤100.3mg/d,

Vitamin E: ≥8.6mg/d VS

≤7.2mg/d

Age, sex

Age, sex, education, number of

siblings ,

Helicobacter pylori infection, vitamin and mineral supplement use, and energy intake

Plummer52 2007

Health questionnaire

/—/ The amount of daily oral/—

follow-up about 3 years

Vitamin A（Beta-carotene 18mg/d ） ,C (750mg/d),E

(600mg/d) VS Placebo

Age, sex, education, area, and smoking

Larsson60,61 2007

FFQ/96/portion size× frequency/Swedish food composition tables

follow-up about 9 years

Vitamin A(Beta-carotene , Retinol):4.25mg/d VS

1.4mg/d, folate: ≥ 260 μ

g/d VS＜203μg/d

Age, sex, education, diabetes, smoking, and energy intake

Carman58 2009

FFQ/124/portion size× frequency/USDA's food composition tables

1 year before interview

Vitamin E:≥7.79mg/d VS

≤5.43mg/d

age, sex, supplementa ry vitamin E, smoking, education, physical

| activity, alcohol use, BMI, and energy intake | | | | |
| --- | --- | --- | --- | --- |
| Pelucchi8 2009 | FFQ/78/ portion size× frequency/Italian food composition tables | — | Vitamin A(Retinol),B1,B2,B6,C,D,E,f  olate  Quartile:(4 VS 1) | Age, sex, education, BMI,  smoking, and  energy intake |
| Neuhouse62 2009 | Health questionnaire  /—/ The amount of daily oral / USDA's food composition tables | follow-up about 8 years | Multivitamins VS Placebo | Age, race, medical history, area, smoking, alcohol use, physical activity, postmenopa usal status,  BMI, and  energy intake |
| Epplein59 2010 | FFQ/81/portion size× frequency/Chinese food composition tables | follow-up about 10 years | Vitamin A: ＞ 0.8mg/d VS  0.46 mg/d ≤ ,Vitamin C:  ＞ 0.11mg/d VS ≤ 0.06 mg/d, Vitamin E: ＞ 16.2 μ g/d VS ≤ 9.5 μ g/d,  folate: ＞ 346.5 μ g/d VS  ≤258.2μg/d | Age, education, smoking, and energy intake |
| Miyazaski5 2012 | FFQ/70/portion size× frequency/Japanese food composition  tables | follow-up about 14 years | Vitamin A: ＞ 1061 μ g/d VS ＜639μg/d | Age, sex |

Abbreviations: BMI: body mass index, FNHDR: Finish National Hospital Discharge Registry, FFQ: food frequency questionnaire, USDA： US Department of Agriculture, USRDA: US Recommended

Daily Allowances.


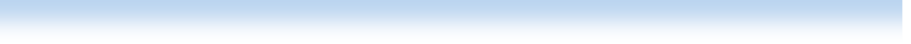


|  | | Selection |  |  | comparability  1 |  |  | Exposure |  | |
| --- | --- | --- | --- | --- | --- | --- | --- | --- | --- | --- |
| Author/year | Adequate | Representativeness | Selection | Definition |  |  | Exposure | Same method | Non-Response | Total |
|  | definition |  |  |  |  |  |  | of |  | quality |
|  | of cases | of cases | of | of |  |  | Ascertainment2 | ascertainment  for all subject | Rate3 | scores |
|  |  |  | controls | controls |  |  |  |  |  |  |
| Correa  1985 | ☆ | ☆ | — | — | ☆☆ |  | ☆☆ | ☆ | — | 7 |
| Risch 1985 | ☆ | ☆ | ☆ | ☆ | ☆☆ |  | ☆ | ☆ | — | 8 |
| You 1988 | ☆ | ☆ | ☆ | ☆ | ☆☆ |  | ☆ | ☆ | ☆ | 9 |
| Buiatti 1990 | ☆ | ☆ | ☆ | ☆ | ☆ |  | ☆☆ | ☆ | ☆ | 9 |
| Boeing  1991 | ☆ | ☆ | — | — | ☆☆ |  | ☆☆ | ☆ | ☆ | 8 |
| Kabat 1993 | ☆ | ☆ | — | — | ☆☆ |  | ☆ | ☆ | ☆ | 8 |
| Ramon 1993 | ☆ | ☆ | ☆ | ☆ | ☆☆ |  | — | ☆ | — | 7 |
| Gonzalez 1994 | ☆ | ☆ | — | — | ☆☆ |  | ☆☆ | ☆ | — | 7 |
| Hansson 1994 | ☆ | ☆ | ☆ | ☆ | ☆☆ |  | ☆☆ | ☆ | ☆ | 10 |
| La Vecchia 1994 | ☆ | ☆ | — | — | ☆☆ |  | ☆☆ | ☆ | — | 7 |
| Cornee 1995 | ☆ | ☆ | — | — | ☆☆ |  | ☆☆ | ☆ | — | 7 |
| Harrison 1997 | ☆ | ☆ | — | — | ☆☆ |  | ☆☆ | ☆ | — | 7 |
| Ji 1998 | ☆ | ☆ | ☆ | ☆ | ☆ |  | ☆☆ | ☆ | ☆ | 9 |
| Garcia-Closas 1999 | ☆ | ☆ | — | — | ☆☆ |  | ☆☆ | ☆ | — | 7 |
| Lopez-Carrilo 1999 | ☆ | ☆ | ☆ | ☆ | ☆☆ |  | — | ☆ | ☆ | 8 |
| Chen 2002 | ☆ | ☆ | ☆ | ☆ | ☆☆ |  | ☆ | ☆ | ☆ | 9 |
| Ekstrom 2000 | ☆ | ☆ | ☆ | ☆ | ☆☆ |  | ☆ | ☆ | — | 8 |
| De Stefani 2000 | ☆ | ☆ | — | — | ☆☆ |  | ☆☆ | ☆ | ☆ | 8 |
| Terry 2000 | ☆ | ☆ | ☆ | ☆ | ☆☆ |  | ☆ | ☆ | ☆ | 9 |
| Jedrychowski | ☆ | ☆ | — | — | ☆☆ |  | ☆☆ | ☆ | — | 7 |
| Mayne 2001 | ☆ | ☆ | ☆ | ☆ | ☆☆ |  | ☆ | ☆ | — | 8 |
| Munoz | ☆ | ☆ | ☆ | ☆ | ☆☆ |  | ☆ | ☆ | ☆ | 9 |

**S4 Table.** Methodological quality of case-control studies included in the meta-analysis.

2001

| 2001 | | | | | | | | | |
| --- | --- | --- | --- | --- | --- | --- | --- | --- | --- |
| Palli  2001 | ☆ | ☆ | ☆ | ☆ | ☆☆ | ☆☆ | ☆ | — | 9 |
| Nomura  2003 | ☆ | ☆ | ☆ | ☆ | ☆☆ | ☆ | ☆ | ☆ | 9 |
| Lissowska  2004 | ☆ | ☆ | ☆ | ☆ | ☆☆ | ☆ | ☆ | — | 8 |
| Qiu  2005 | ☆ | ☆ | ☆ | ☆ | ☆☆ | ☆ | ☆ | ☆ | 9 |
| Lunet  2006 | ☆ | ☆ | ☆ | ☆ | ☆☆ | ☆ | ☆ | — | 8 |
| Kim  2005 | ☆ | ☆ | — | — | ☆☆ | ☆☆ | ☆ | — | 7 |
| Pelucchi  2009 | ☆ | ☆ | — | — | ☆☆ | ☆☆ | ☆ | — | 7 |

1A maximum of 2 stars could be assigned for this item. Studies that controlled for age received one star, whereas studies that controlled for intake of other nutrients received an additional star. 2A maximum of 2 stars could be awarded for this item. Studies that ascertained the expose with secure record or structured interview blind to case/control status received one star, whereas studies that considered the issues related to dietary changes because of disease received an additional star. 3One star was assigned if there was no significant difference in the response rate between case and control subjects by using the chi-square test. (*P* > 0.05)

# **S5 Table.** Methodological quality of cohort studies included in the meta-analysis

| Author |  | Selectio n |  |  | Compara bility1 |  |  | Outco me |  | Tota l |
| --- | --- | --- | --- | --- | --- | --- | --- | --- | --- | --- |
| /year | Representati veness of the  exposed | Selectio n of the non-exp osed | Ascertain ment of  exposure | Outco me of intere st not prese nt  at |  |  | Assess ment of  outcom | Follow  -up long enoug h for out  comes | Adequ acy to  follow up of  cohort | qual ity  scor |
|  | cohort | cohort |  | start of study |  |  | e | to occur2 | 3 | es |
| Larsson  2007 | ☆ | ☆ | ☆ | ☆ | ☆ |  | ☆ | ☆ | ☆ | 8 |
| Botter  weck  2000 | ☆ | ☆ | ☆ | ☆ | ☆ |  | ☆ | ☆ | ☆ | 8 |
| Carma | ☆ | ☆ | ☆ | ☆ | ☆☆ |  | ☆ | ☆ | ☆ | 9 |

| n  2009 | | | | | | | | | |
| --- | --- | --- | --- | --- | --- | --- | --- | --- | --- |
| Epplein  2010 | ☆ | ☆ | ☆ | ☆ | ☆ | ☆ | ☆ | ☆ | 8 |
| Miyaza ski 2012 | ☆ | ☆ | ☆ | ☆ | ☆ | ☆ | ☆ | ☆ | 8 |
| Neuho user 2009 | ☆ | ☆ | ☆ | ☆ | ☆ | ☆ | ☆ | ☆ | 8 |
| Zheng  1995 | ☆ | ☆ | ☆ | ☆ | ☆ | ☆ | ☆ | ☆ | 8 |

1A maximum of 2 stars could be awarded for this item. Studies that controlled for age received one star, whereas studies that controlled for intake of other nutrients received an additional star. 2A cohort study with a follow-up time > 5 years was awarded one star.

3A cohort study with a follow-up rate > 80% was awarded one star.

# **S6 Table.** Methodological quality of RCTs included in the meta-analysis.

| Trial | Generaltion of the  allocation sequence | Allocation concealm ent | Blinding | Follow-up | Samp le size cacul ation | Inten tion-t o-tre at analy  sis | Methodo logical quality |
| --- | --- | --- | --- | --- | --- | --- | --- |
| NIT2 1993 | Adequade | Adequade | Adequade | Adequade | Yes | Yes | High |
| PHS 1996 | Adequade | Adequade | Adequade | Adequade | Yes | Yes | High |
| Correa 2000 | Adequade | Adequade | Adequade | Adequade | No | No | High |
| HPS 2002 | Adequade | Adequade | Adequade | Adequade | Yes | Yes | High |
| ATBC 2003 | Adequade | Adequade | Adequade | Adequade | Yes | Yes | High |
| Zhu 2003 | Unclear | Unclear | Adequade | Adequade | Yes | No | Low |
| CARET 2004 | Adequade | Adequade | Adequade | Adequade | Yes | Yes | High |
| SUVIMAX 2004 | Adequade | Adequade | Adequade | Adequade | Yes | Yes | High |
| WHS 2005 | Adequade | Adequade | Adequade | Adequade | Yes | Yes | High |
| SIT 2006 | Adequade | Adequade | Adequade | Adequade | Yes | Yes | High |
| Plummer 2007 | Adequade | Adequade | Adequade | Adequade | No | Yes | Low |

**S7 Table.** Dose-response analysis.

| Type of | Number | Dose | RR（95%） |  | Heterogeneity test | |
| --- | --- | --- | --- | --- | --- | --- |
| vitamins | of studies | increment |  | χ*2* | *P* | I2(%) |

| Vitamin A | 8 | 1.5 mg/d | 0.71(0.62,0.81) | 9.03 | <0.0000  1 | 22 |
| --- | --- | --- | --- | --- | --- | --- |
| Vitamin C | 11 | 100mg/d | 0.74(0.69,0.79) | 10.42 | <0.0000  1 | 4 |
| Vitamin E | 8 | 10mg/d | 0.76(0.67,0.85) | 12.24 | <0.0000  1 | 43 |
| Vitamin D | 2 | 1.4mg/d | 1.27(1.06,1.51) | 0.28 | 0.009 | 0.0 |
| Vitamin B1 | 2 | 1.3mg/d | 0.83(0.51,1.35) | 1.99 | 0.44 | 50 |
| Vitamin B2 | 2 | 1.4mg/d | 0.71(0.35,1.46) | 4.62 | 0.35 | 78 |
| Vitamin B6 | 2 | 1.2mg/d | 0.85(0.62,1.17) | 0.88 | 0.32 | 0 |
| Vitamin M  (Folate) | 7 | 260ìg/d | 0.85(0.70,1.04) | 22.69 | <0.0001 | 74 |

**S8 Table.** Meta-regression analysis.

| Variable | Coefficient | Standard error | *P* value | 95% CI |
| --- | --- | --- | --- | --- |
| Study design | 1.009 | 0.057 | 0.075 | 0.990 - 1.220 |
| Geographic area | 0.999 | 0.070 | 0.992 | 0.867 - 1.152 |
| Doses of vitamin | 0.656 | 0.948 | 0.006 | 0.491 - 0.878 |
| Vitamin intake assessment method | 1.201 | 0.076 | 0.006 | 1.056 - 1.365 |

**S9 Table.** Tolerable upper intake levels of vitamins.

| Life stage | Vitamin A  ìg/d | Vitamin B6  mg/d | Folate  ìg/d | Vitamin C  mg/d | Vitamin D  ìg/d | Vitamin E  mg/d |
| --- | --- | --- | --- | --- | --- | --- |
| Male (19-50 y) | 3000 | 100 | 1000 | 2000 | 100 | 1000 |
| Female (19-50 y) | 3000 | 100 | 1000 | 2000 | 100 | 1000 |
