# Supplementary figures and images for: Vitamin Intake Reduce the Risk of Gastric Cancer: Meta-Analysis and Systematic Review of Randomized and Observational Studies
Source: PLoS One. 2014 Dec 30;9(12):e116060. doi: 10.1371/journal.pone.0116060 (PMC4280145; doi:10.1371/journal.pone.0116060)

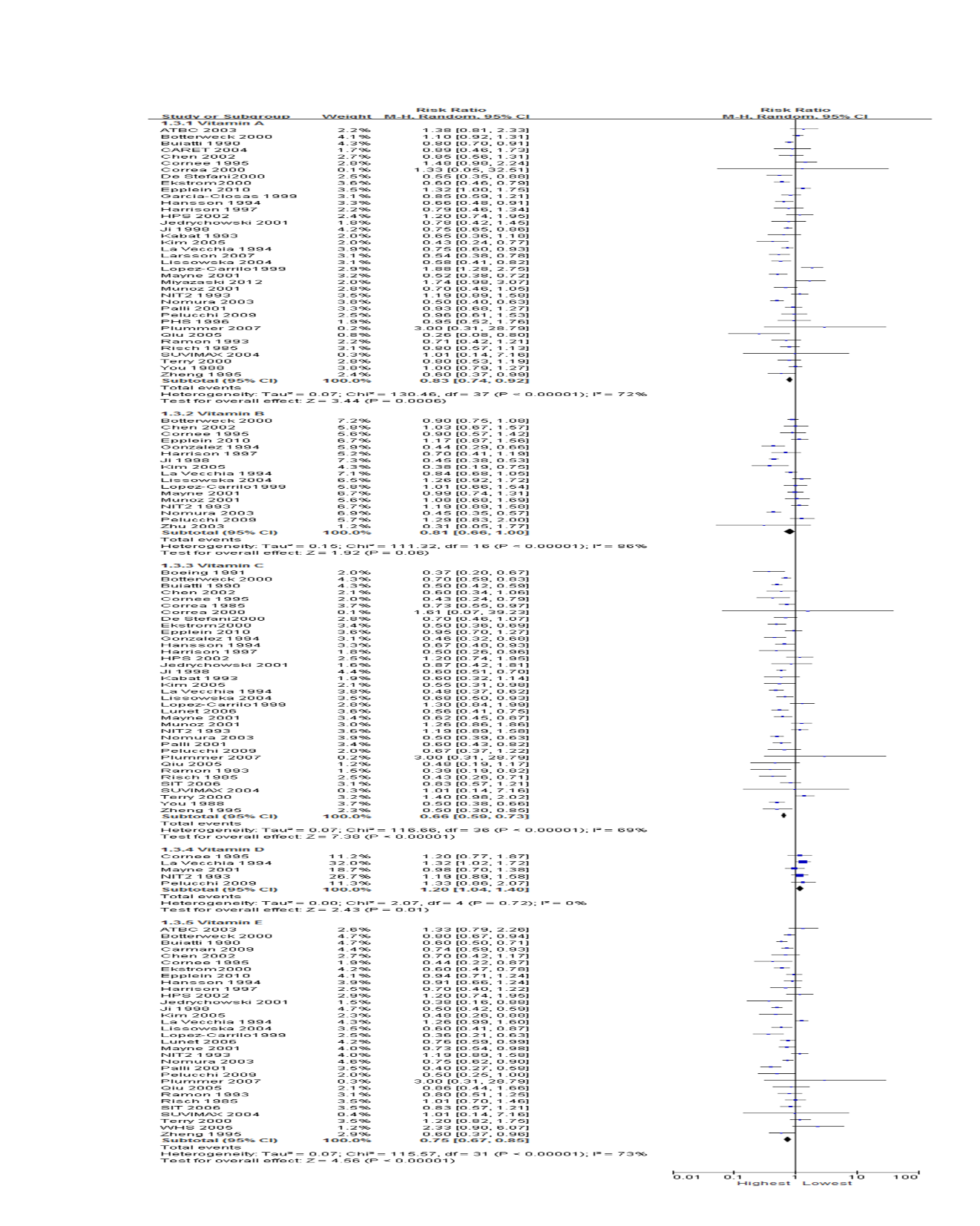


**S1 Figure.**

**
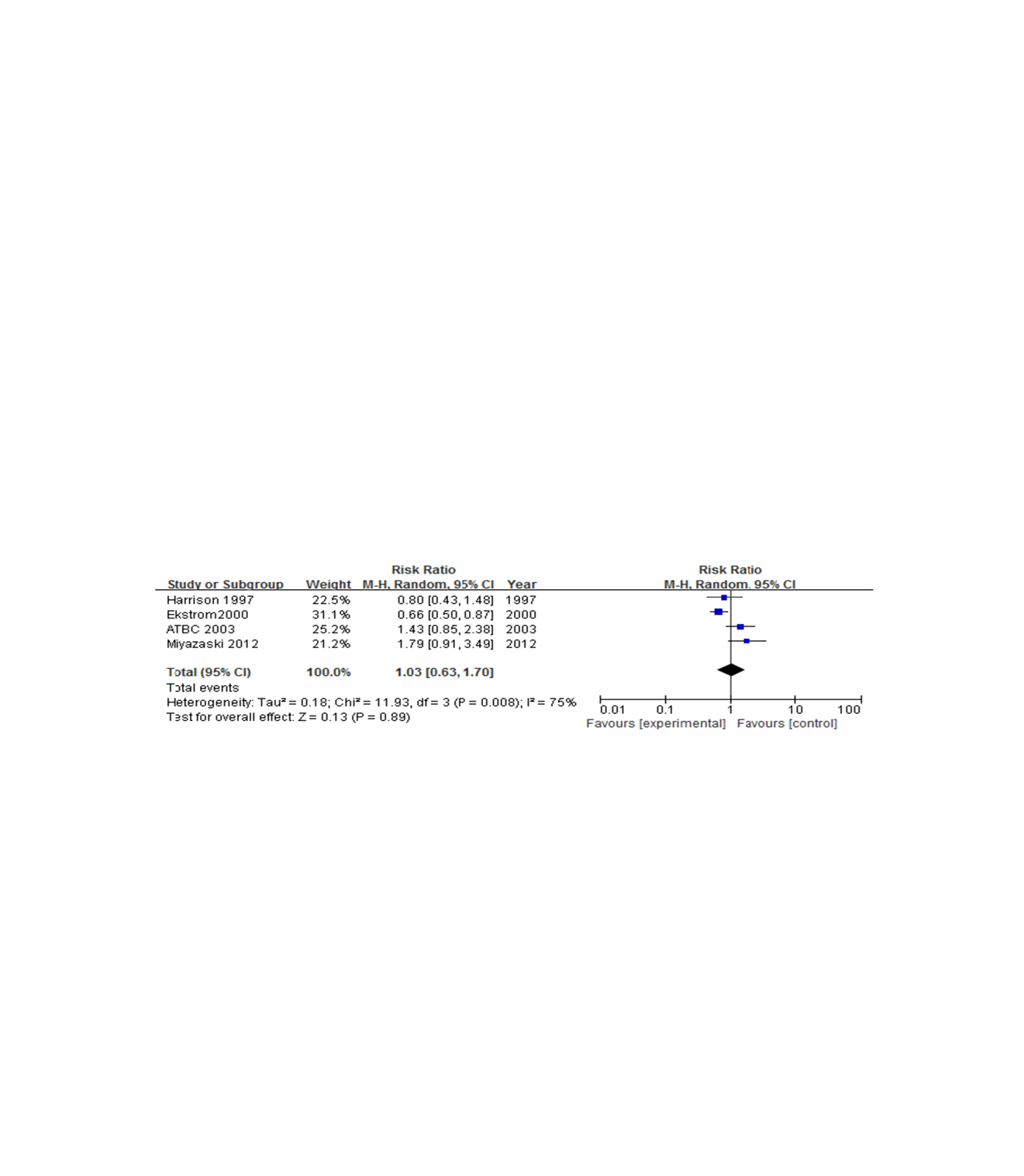
**

**S2 Figure.**


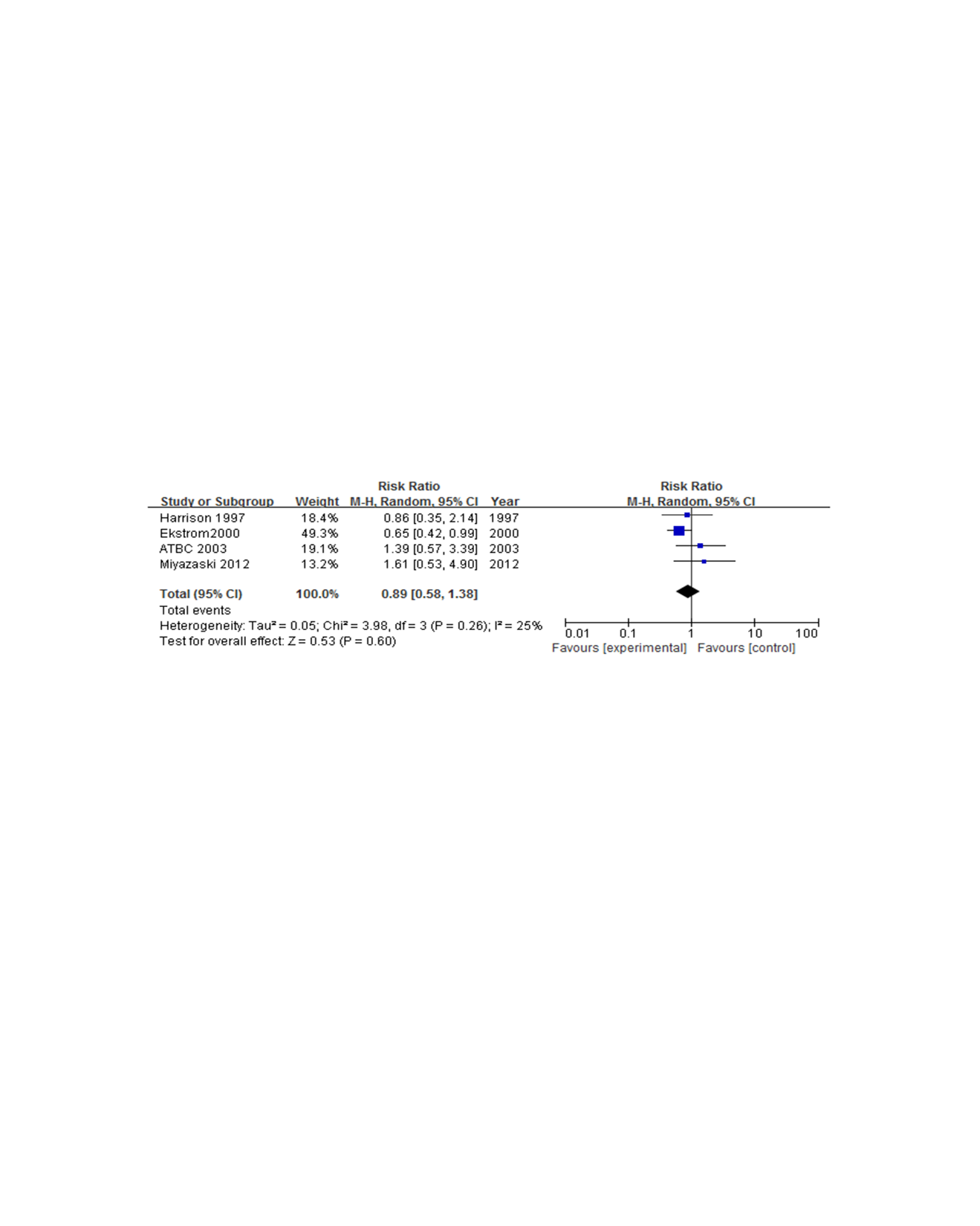


**S3 Figure.**


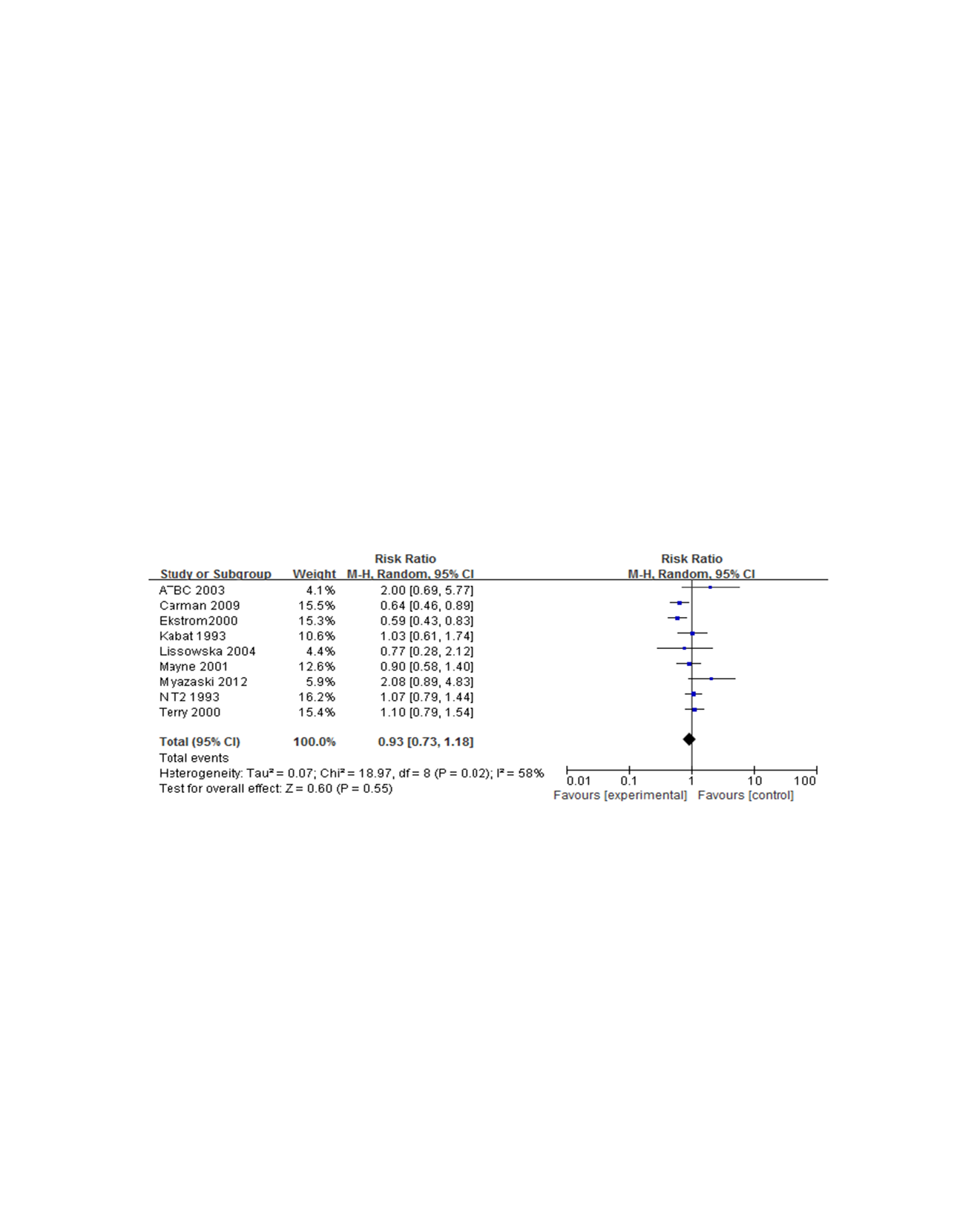


**S4 Figure.**


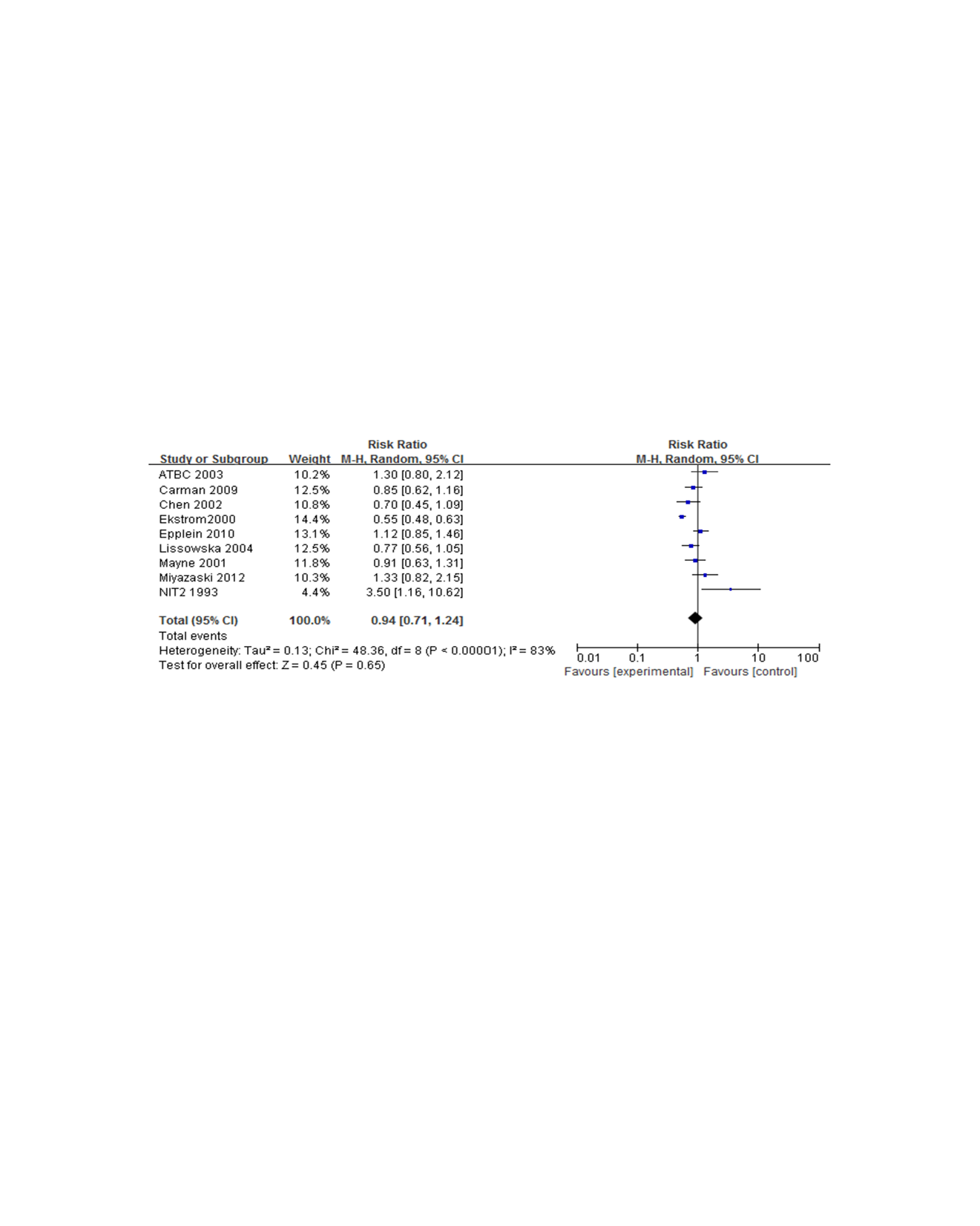


**S5 Figure.**


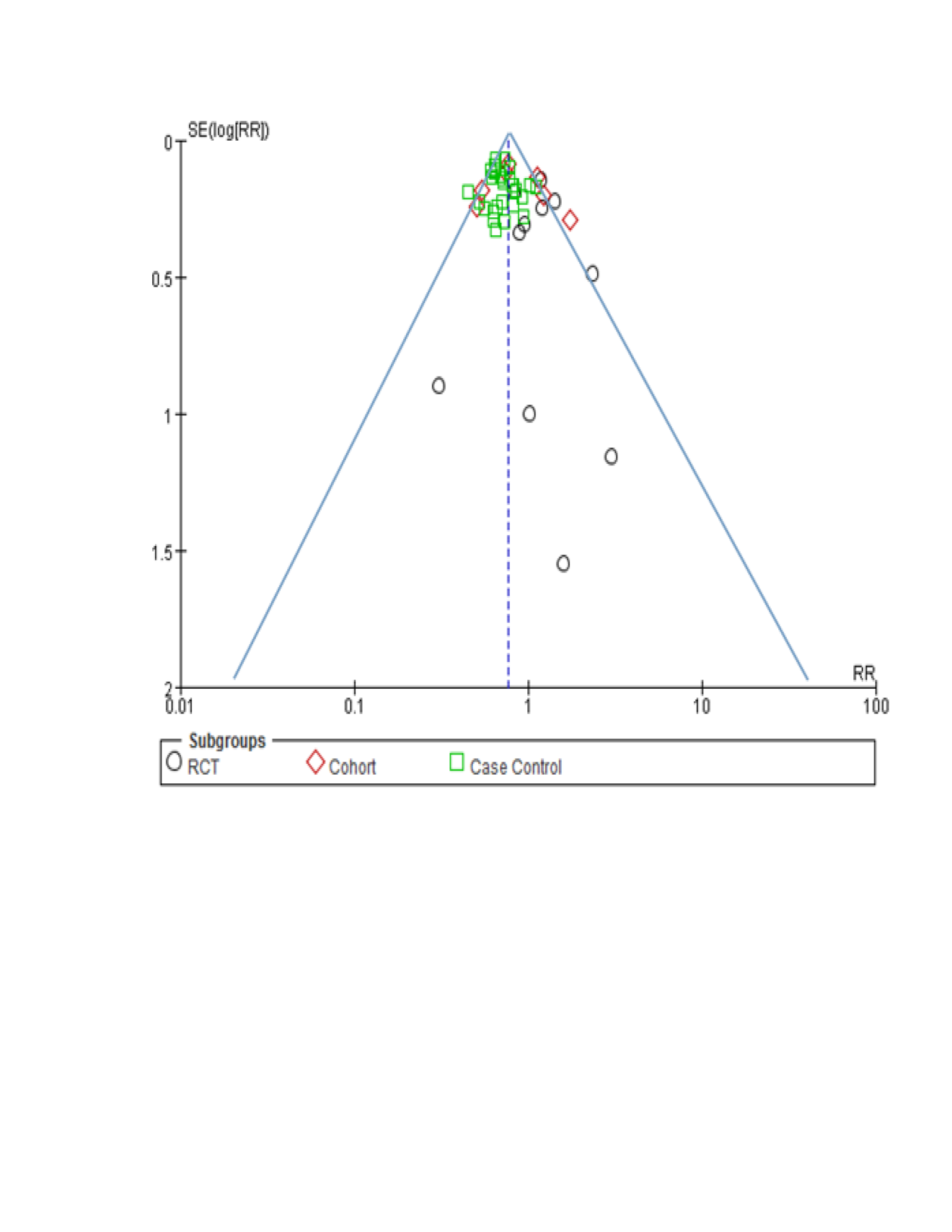


**S6 Figure.**

Supplement: S2 File — Supporting Information Figures. S1 Figure. Subgroup analysis: Forest plot of vitamin type. CI, confidence interval; df, degrees of freedom; I2, the percentage of total variation across studies that is caused by heterogeneity rather than by chance Squares or diamonds to the left of the solid vertical line indicate benefit with each type of vitamin intake; this is conventionally significant (P<0.05) only if the horizontal line or diamond does not overlap the solid vertical line. Relative risks are analysed with random-effects model. S2 Figure. Subgroup analysis: Forest plot of Lauren’s classification (intestinal). CI, confidence interval; df, degrees of freedom; I2, the percentage of total variation across studies that is caused by heterogeneity rather than by chance. S3 Figure. Subgroup analysis: Forest plot of Lauren’s classification (diffuse). CI, confidence interval; df, degrees of freedom; I2, the percentage of total variation across studies that is caused by heterogeneity rather than by chance. S4 Figure. Subgroup analysis: Forest plot of location (cardia). CI, confidence interval; df, degrees of freedom; I2, the percentage of total variation across studies that is caused by heterogeneity rather than by chance. Relative risks are analysed with random-effects model. S5 Figure. Subgroup analysis: Forest plot of location (non-cardia). CI, confidence interval; df, degrees of freedom; I2, the percentage of total variation across studies that is caused by heterogeneity rather than by chance. Relative risks are analysed with random-effects model. S6 Figure. Funnel plot of included studies. RCT: Randomized, Placebo-Controlled Trial. The oblique line in the center is the natural logarithm of pooled relative risk, and the 2 solid lines are pseudo 95% confidence limits. (DOCX) [file pone.0116060.s003.docx]
